# Profiling Age of Males Exposed to Poisoning and Outcomes at a Tertiary Hospital

**DOI:** 10.1002/alz70860_107159

**Published:** 2025-12-23

**Authors:** Vita Mithi

**Affiliations:** ^1^ Kamuzu University of Health Sciences, Blantyre, Blantyre, Malawi

## Abstract

**Background:**

The African health sector continue to pay little attention to understand concentrations and effects of different poisons in age groups. Meanwhile, age and being male is considered as a risk for neurodegenerative disorders such as dementia in mid‐life. Thus this study, aims to summarize the ages of males exposed to different poisons diagnosed at a tertiary hospital in northern Malawi to address and initiate the need for robust epidemiological, biomedical neuroscience data and novel tests for poison dementia identification, health outcomes and disease variations.

**Method:**

Retrospective data of hospitalized males, diagnosed of poisoning, from January 2022 and December 2023 at Mzuzu Central Hospital was collected and analyzed following the world health organization age group standardization. Descriptive analysis included the diagnosed poison, age, length of hospital stay, and outcomes.

**Result:**

From 208 cases of poisoning diagnosis, alcohol intoxication (*n* = 136, 65%) was highest, in 25 to 29 (*n* = 29, 13.94%) age group, followed by 30 to 34 (*n* = 26, 12.50%), 35 to 39 (*n* = 21, 10.10%), 40 to 44 (*n* = 19, 9.13%), and 20 to 24 (*n* = 18, 8.65%). Organophosphate poisoning accounted for 19.71% (*n* = 41), common in 25 to 29 (*n* = 12, 5.77%) and 30 to 34 (*n* = 8, 3.85%) age groups. General poisoning was 2.40% (*n* = 5), in persons aged 20 to 24 years. Meanwhile food poisoning was 6.25% (*n* = 13), sharing equal proportion of 1.92% (*n* = 4) in 15 to 19 and 20 to 24 ages respectively. Drug poisoning was in 40 to 44 age group (*n* = 1, 0.48%). Interestingly, alcohol intoxication in dementia risk age group of 65 to 69 was 0.96% (*n* = 2), sharing proportion in 70 to 74 (*n* = 1, 0.48%) age group for organophosphate poisoning. Median age of the patients was 31 years (range: 15‐71years) with a hospital stay of 3 days (interquartile range 2–4 days). Regarding outcome, there was no follow up on discharge patients (93.27%), those that left without completing treatment and physicians’ knowledge (1.44%), and (5.29%) that died while admitted.

**Conclusion:**

The study shows that adolescents and middle aged adults are highly exposed to substances that induce electrolytes imbalance and make the brain vulnerable to young onset of poison dementia.

